# Urinalysis*Read at meeting of the Austin District Medical Association, December 21, 1899, Austin, Texas.

**Published:** 1900-01

**Authors:** W. R. Neville

**Affiliations:** Austin, Texas


For Texas Medical Journal.

### Urinalysis.*

BY W. R. NEVILLE, PH. D., AUSTIN, TEXAS.

The subject chosen is one that the practitioner is called upon to face frequently (in some localities and instances it is of daily occurrence), but to correctly diagnose and differentiate is another thing.

Ordinarily, upon the examination of a patient where kidney trouble is suspected, it has been the usual custom to secure a small portion of the urine passed at that time, or, again, it may be a sample passed either before retiring or upon rising; it is then taken to the office and usually the old method of examining for albumen and sugar, the former by the heat and nitric acid test and the latter by Fehling's or Trommer's. In the former instance, unless the urine is heavily loaded with albumen and the acid coagulates and the heat precipitates it immediately, the diagnosis is usually formed at once, when it is clearly proven by the majority of works on this subject, including Purdy, Tyson, Stern, Yarbrough and others that after testing, the urine should be allowed to stand several hours.

After a careful study of this subject, and making hundreds of examinations, I am of the firm opinion that to examine urine properly, a specimen should be obtained from all the urine passed in twenty-four hours; my authority for saying this, outside of the authors quoted above, has been from personal observation in examining specimens taken from the same patient at various hours of the day. For example, will cite a late case, which was examined as morning urine and after the most rigid test, and allowing to stand the proper time, no trace of albumen was found. Then later in the day sample from same patient showed albumen very soon after applying the test.

All physicians may not be prepared to make as thorough an examination as the case demands, even should time permit. The real busy practitioner has not the time to make examinations as they should be; it is frequently inconvenient to test the urine when received, and sometimes a day or two passes before he thinks of it.

It is useless to take up your time in this paper, calling your attention to the color, acidity, normal and abnormal constituents, but

*Read at meeting of the Austin District Medical Association, December 21, 1899, Austin, Texas.

will pass on to the more important—test. The specific gravity is taken to ascertain the relative amount of solids contained in the urine, and here is an important point—the urinometers on the market are of various makes and tested at various temperatures. Some mark them to examine the urine at 60 degrees, others at 70 degrees and 75 degrees, and they accompany the urinometer with a thermometer to take the temperature. As a usual thing the urinometer reads from 0 to 50 or 60, the reading of urine. Showing a gravity of 20 by urinometer would be read as 1020, water, 1000.

If the amount of urine received is small and it is necessary to examine at once and not wait for another sample, it may be diluted with one or two volumes of water, carefully measured, and the "specific gravity" of this dilution taken. The figures as read from the urinometer must then be multiplied by the number of volumes of water used, plus one. For example, we have one-half ounce of urine and have to dilute it to two ounces to get the specific gravity, which is found to be 1006. We have used one and one-half ounces of water or three volumes of water to one of urine; now multiply the six by the number of volumes of water plus one and add to the amount 1000, which will give 1024, the specific gravity desired.

Amongst the normal constituents, examined for are "uric acid," by the murexide test, reagent nitric acid and dilute ammonia; mucin is detected by acetic acid and liquor iodine comp. Hippuric acid, reagent nitric acid; sulphates, test by barium chloride solution. Until recently the proteines occurring in the urine were all classed as albumen. There are at least six proteine substances which may appear in the urine, each of different significance; the one interesting used mostly is serum albumen.

Serum albumen occurs in urine mostly with paraglobules and in minute quantities one per cent. to two per cent. by actual weight. The appearance of albumen in the urine does not indicate renal changes (but may be traced to albuminous foods) unless accompanied by cast, epithelium, etc.

Detection of albumen may be made by heat. Add a few drops of acetic acid to test tube two-thirds full; boil upper part of urine; if it looks turbid where heated, let it cool, afterwards add a few drops nitric acid, if turbidity remains or increases albumen is present.

Place about two drachms of suspected urine in test tube and boil; if precipitate occurs it is due to albumen or earthy phosphates. To differentiate add a few drops of nitric acid. If precipitate disappears it is due to presence of earthy phosphates (test for same with the magnesium fluid, which will be given later). See addendum. If it remains, it is caused by albumen.

Hiller's test is to place an inch of nitric acid in test tube and drop the same amount of urine, drop by drop, along the side of the tube. This is necessary, as the urine must lay on the top of the acid and not mix; notice opalescent zone at point of contact. If small traces of albumen are present, it may take one-half to three-fourths hour before the ring appears. If mucin be present in excess a light turbidity may appear near the surface when this test is applied.

Purdy's test: Raise the specific gravity of urine ten to fifteen degrees by addition chloride solution, fill test tube two-thirds full, add one or two drops strong acetic acid and boil upper part of urine half a minute. If albumen be present it will appear in the upper boiled portion and the lower portion will remain clear.

The Ferrocyanide test is very similar to Prof. Elliott's (of Chicago) modified test, which is prepared with

| | |
|---|---|
| Iodide potassium | 3.32 grams. |
| Bi-chloride mercury | 1.35 grams. |
| Acetic acid | 20 c. c. |
| Distilled water | 64 c. c. |

Dissolve the potassium and mercury separately in water, mix the solution, then add acetic acid and filter. Fill test tube half full of urine, add five to ten drops of acetic acid and one drachm of the reagent. If albumen be present in the smallest amount, a precipitate will occur. If precipitate occurs,—heat if caused by peptons,—it will disappear or diminish. If by serum albumen, or by mucin, it will remain unaltered or will be intensified.

Esbach's picric acid method is used mostly as a percentage test. His solution is prepared by dissolving one gram. picric acid, two grams. citric acid in one hundred grams. of water, using a graduating test tube, which is filled half urine, half reagent, and mixed thoroughly, allowed to stand twenty-four hours and the precipitate is read in percentage instead of grammes.

Peptons often occur in combination with albuminose and closely resemble; differentiate, peptons, gives no precipitate with nitric acid—is not precipitated with ammonium sulphate, while albuminose is precipitated. Saturate slightly acidified urine with ammonia sulphate and filter out any precipitate which may consist of albumen, globuline, and albuminose. Proteines remaining may be precipitated by potassa mercuric iodide and can be only pepton.

"Dextrose," "Glucose," "grape sugar," occurs in urine as a result of temporary condition. As a general rule, the urine has high specific gravity. Whenever urine shows a specific gravity above 1028, should be tested for dextrose—which may be done by Boettgart's bismuth test—"liq. potassa and sub nit bismuth." The albumen should always be removed before applying the test. (It is claimed that certain medicine, viz.: turpentine, capabia will turn the bismuth black.) We then have recourse to Fehling's, which should always be prepared fresh. We have also Trommer's—the picric acid and others, but Haine's is the most reliable, even surpassing the yeast or fermentive. There is one thing to be borne in mind in testing for dextrose with any test, that is, we may so easily be mistaken and should bear in mind that nitric acid will not dissolve sugar, but other sediment that is sometimes taken for sugar. We should always have a weak solution of honey or glucose to try, for show up, as a correspondent test. One drop of honey or glucose in eight ounces of water will give a marked reaction with Haine's test.

Phenal hydrazin test is one used microscopically. It is said that this test is trustworthy and can be highly recommended as it gives no reaction with any other substance of the urine than grape sugar. It is a very difficult test to make and requires from two to three hours to complete. The yeast test requires over twenty-four hours to complete and the temperature to be maintained at 80 degrees and two separate samples of same urine. Dr. Whitney, of New York, has adopted a volumetric test for sugar which is a modification and addition of Fehling's and said to be accurate and simple except so far as making this solution, which, by the way, can be bought.

Acetone in the urine may be detected by Chantaid's test or Leiben's test. Pus in urine is increased by heat; acid dissolves the phosphates and increases the turbidity. Bile and bileary acids are detected by Pettenkofe's test—"conc. sul. acid and cane sugar."

The microscope is a valuable adjunct in urinalysis and this can never be called complete, unless the sediments are examined. The sediments are usually obtained by allowing the urine to settle or by means of centifuge. The necessary technique can only be acquired by practical instructions.

Urinary sediments may be divided into two classes—chemical bodies, and anatomical bodies, viz.: uric acid, acid urate of soda, potassium and lime, calcium oxalate, calcium sulphate, etc. Triple phosphates—phosphate calcium, ammonia Leucein, trystin, etc.

Anatomical bodies consist of, or contain blood, pus, epithelial cells, cast, fungi, bacteria and spermatozoa and at some certain time it may be necessary to examine for one or more of these articles in urine to complete a diagnosis. On one occasion I was employed by a lawyer to examine a specimen of urine with instructions to examine carefully and thoroughly, and I found, amongst other things, spermatozoa. Fortunately I was not called into court, but think the case in question went through the courts.

It is necessary in testing urine to be sure all your reagents are pure, as usually, those found in the majority of drug stores, the solutions and chemicals, are not to be relied upon. I am now using, and have been for some time, solutions made from Merck's C. P. chemicals, and I find upon close investigation he has two or three grades, and I only use chemical pure guaranteed reagent and obtain better results.

In studying the different reaction of the various tests, I have come across instances in testing for sugar where the yeast test was used. It failed to ferment, which I ascertained was due to certain internal medicines that had been administered—quinine and salol. Salicylic acid and salicylate of soda, when taken for several days, will arrest fermentation and though sugar may be present it will not show unless the aqua sappherina (Wilthan's) is used. Elirlich's Diazo Reaction, introduced in 1882, as a test for typhoid fever has not proved successful in my hands. If it becomes necessary to send away a sample of urine for examination or keep for several days it will be best to add a preservative to it, and the best thing I know of is Thymol, as it does not interfere with any other constituent of the urine.

ADDENDUM.

Formula for magnesia fluid for testing for earthy phosphates:

℞ Liquor ammonia....................1 part.
Ammonia chloride....................1 part.
Magnesia sulphate....................1 part.
Distilled water....................8 parts.

M.

Filter off the precipitated earthy phosphates (precipitated by the addition of sodium, potassium and heat) and add to the filtered urine one-third its volume of the reagent snowy deposits—precipitated alkaline phosphates. Examined under the microscope will show most beautiful crystals.

